# Unveiling the Narratives of Portuguese Professionals Engaged with Domestic Violence Victims: Persistent Challenges and Novel COVID-19 Impacts

**DOI:** 10.1177/08862605241262243

**Published:** 2024-07-27

**Authors:** Elisabete Ferreira, Ana Sofia Figueiredo, Anita Santos

**Affiliations:** 1 University of Maia, Portugal; 2 University of Porto, Portugal

**Keywords:** domestic violence, intervention challenges, professional narratives, guidelines, qualitative research, COVID-19 pandemic, crisis preparedness

## Abstract

Domestic violence remains a complex and challenging issue, particularly for professionals engaged in providing support to victims. The occurrence of emergency situations, such as the COVID-19 pandemic, further exacerbates the difficulties faced by these practitioners. This study aims to explore the lived experiences of professionals working with domestic violence victims, with a specific focus on the primary challenges encountered during interventions. It also aims to identify key guidelines that could enhance their practices. Twenty-four professionals from the domestic violence victim support in Portugal participated in this qualitative research. Through semi-structured interviews and thematic analysis, the study identified a range of challenges professionals confront in their intervention efforts. These challenges encompassed various aspects of the intervention process, vulnerabilities observed in victims, and the existing support system. Furthermore, the study uncovered specific challenges posed by the COVID-19 pandemic. Alongside these challenges, the research highlighted a set of recommendations designed to refine intervention strategies and promote better professional adaptation. The findings underscore the array of challenges that professionals grapple with, impacting both their strategies for intervention and their overall well-being. Thus, the development of effective intervention methodologies for professionals and organizations emerges as a crucial endeavor, essential for assisting domestic violence victims in their daily lives and enhancing preparedness for potential future crises.

The Council of Europe Convention on Preventing and Combating Violence Against Women and Domestic Violence (Council of Europe, 2011), known as the Istanbul Convention, is a crucial legal instrument defining Domestic Violence (DV). Ratified by the Portuguese State in 2013, the convention defines DV as various forms of violence occurring within the family or domestic unit, involving current or former partners, regardless of shared domicile. This comprehensive definition encompasses intimate partner violence (IPV), abuse of children and adolescents, and victimization of the elderly within familial settings (Ribeiro et al., 2022). DV is a pervasive social issue, affecting individuals from diverse backgrounds regardless of gender, nationality, or education. The European Union’s survey on violence against women, encompassing 42,000 women from 28 European states, has revealed alarming statistics, with 22% of women reporting being victims of physical and/or sexual violence by an intimate partner, and 43% enduring some form of psychological violence (European Union Agency for Fundamental Rights [FRA], 2014).

In Portugal, DV remains a prevalent issue, as it accounted for the highest number of reported crimes in 2022. The Portuguese police authorities received a total of 30,488 reports, representing a 15% increase compared to the previous year (SSI, 2023). According to the same source, most of these reported incidents (86%) involved violence against spouses or similar partners, with women comprising 72.4% of the victims and men representing 80.2% of the reported offenders. Additionally, within the DV context, there were 28 reported cases of homicide, which marked an increase of 5 compared to the previous year (SSI, 2023).

A recent study conducted by Neves et al. (2022) in Portugal aimed to characterize death threats and attempts of femicide from the perspectives of professionals within the National Support Network for Victims of Domestic Violence (RNAVVD), contributing to best practices in risk assessment. The study involved 71 professionals who completed an online questionnaire survey via Google Forms. The findings revealed that the majority of professionals (98.6%) reported working with female victims. These victims are primarily between the ages of 26 and 60, involved in heterosexual intimate relationships, with 60.6% of the professionals indicating that many of these victims are married. Additionally, 94.4% of the professionals reported that these victims are mothers, with 85.9% stating that most of the children are under the age of 18. The study also indicated that DV manifests in multiple forms, including psychological, physical, economic, social, and sexual abuse, and is predominantly perpetrated within intimate relationships, as reported by 95.8% of the professionals.

The complex and devastating nature of DV has prompted the development of an extensive network of resources, including hotlines, shelters, and community-based services, aimed at supporting and protecting DV victims and their children (Goodman et al., 2020). Consequently, particular attention has been directed toward understanding and addressing the primary challenges and difficulties faced by professionals operating in this domain (Burnett et al., 2016; Caridade & Sani, 2018; Kulkarni et al., 2012; Merchant & Whiting, 2015; Radey & Stanley, 2018; Tavormina & Clossey, 2017). Intervening with DV victims is widely acknowledged as a complex and multifaceted practice, enclosing challenges that stem from diverse sources, including the professionals’ own skills and capabilities (Caridade & Sani, 2018) as well as societal, workplace, and client-related factors (Merchant & Whiting, 2015). In a qualitative investigation, Kulkarni et al. (2013) examined the experiences of 30 DV victims and 24 hotline advocates. The study revealed that professionals identified several factors influencing DV victim intervention, including inadequate organizational resources, staff burnout, lack of training, and poor integration with other community resources. Additional challenges were attributed to bureaucratic obstacles, high caseloads, and cultural tendencies to downplay or deny the existence of DV (Frieze et al., 2020; Kulkarni et al., 2013; Radey & Stanley, 2018). Similarly, Merchant and Whiting (2015) studied critical factors affecting DV intervention, including the emotional toll of client stories, handling crisis situations, addressing clients’ return to abuse, and promoting empowerment. Participants also highlighted the challenges in meeting diverse needs of DV victims in their intervention. Additional literature corroborates this complexity, highlighting the difficulty of effectively addressing the DV victims’ multifaceted needs, which include safety concerns, economic challenges, substance use, mental health issues, and uncertainties about life changes (Arroyo et al., 2017; Burnett et al., 2016; Sullivan & Goodman, 2019). This limitation in effectively addressing the victims’ needs can often lead, as reported by professionals, to feelings of frustration and powerlessness among them (Tavormina & Clossey, 2017). Additionally, professionals working with DV victims frequently encounter elevated levels of stress arising from direct and indirect exposure to the victims’ narratives and distress (Kulkarni et al., 2013). For instance, Benuto et al. (2018) conducted a quantitative investigation involving 135 victim advocates, uncovering a substantial occurrence of Secondary Traumatic Stress at around 50%. In a separate study, Ellis and Knight (2021) conducted an ethnographic exploration of victim service providers, who reported a negative impact, evident through intrusive memories, disrupted sleep patterns, or heightened anxiety.

Despite substantial research on the challenges faced by professionals working with DV victims, a notable gap exists in understanding how these challenges intersect with the unparalleled influence of the COVID-19 pandemic. The global crisis triggered by the pandemic required rapid adjustments in interventions with DV victims, leading to increased use of innovative technologies and remote support methodologies not widely employed before (Ghidei et al., 2022; Macy, 2022; Voth Schrag et al., 2023; Wright et al., 2022). Remote working arrangements became prevalent, although not all organizations were adequately prepared to implement such changes (van Gelder et al., 2021). Recent empirical research supports these shifts, as studies have reported a significant increase in remote interventions and a reduction in face-to-face (f2f) interactions (Macy, 2022; Ribeiro et al., 2022; Voth Schrag et al., 2023; Wright et al., 2022). Similar findings were observed in a cross-sectional study conducted in Portugal (Caridade et al., 2021), which revealed that 57.7% of professionals suspended f2f support, and telephone emerged as the primary remote communication medium used during lockdown. Additionally, a qualitative study suggested that these changes prompted a shift in the approach toward victims, favoring a more reactive and crisis-oriented stance, in contrast to the earlier proactive approach (Pfitzner et al., 2020).

Although some research has explored the pandemic’s impact on DV victims, limited attention has been given to professionals’ experiences within this context (van Gelder et al., 2021). As frontline workers, professionals supporting DV victims had to swiftly adjust their practices to meet victims’ needs while managing personal, familial, and workplace impacts of the pandemic (Garcia et al., 2022; Self-Brown et al., 2022). In a qualitative study involving 53 IPV advocates working with DV victims during the COVID-19 pandemic, various challenges emerged, including mental health symptoms, reduced colleague interaction, and adapting to remote work (Garcia et al., 2022). Similar difficulties were also documented in other research, which highlighted impediments like establishing empathetic relationships, limited victim access, technological constraints, boundary maintenance, and difficulties in achieving work-life balance (Voth Schrag et al., 2023; Wood et al., 2022). Additional investigation by Van Gelder et al. (2021) revealed that professionals encountered feelings of frustration, insecurity, and loneliness due to changes in their work conditions. A cross-national quantitative study conducted in the United States, Canada, and Australia found that COVID-19 significantly influenced professionals’ daily lives, with many assuming increased caregiving responsibilities for children or ill family members (Self-Brown et al., 2022). Furthermore, changes in service delivery methods have been linked to heightened clinician stress (Pfitzner et al., 2020), and approximately 40.2% of participants reported feelings of overwhelm, anxiety, stress, depression, or worry during this period, according to Self-Brown et al. (2022).

## Present Study

Despite the presence of some studies investigating DV in the Portuguese context (Caridade et al., 2021; Pérez et al., 2023), to the best of our knowledge, no research in Portugal has specifically explored the perspectives of professionals regarding the main challenges and the impact of their work with DV victims, especially in the context of the unprecedented COVID-19 pandemic. Therefore, the present study aims to fill this research gap by comprehensively understanding the experiences and narratives of professionals engaged with DV victims. This endeavor aims to uncover the primary challenges they face when intervening with victims and gather their insights on how to improve interventions. It is essential not only to explore the usual difficulties in these interventions but also to understand how the COVID-19 pandemic has introduced new challenges and obstacles. By doing so, the study aims to provide practical recommendations based on their experiences to enhance DV support services, especially in times of crisis like the COVID-19 pandemic.

## Method

The present research aims to understand the lived experiences of professionals dealing with DV through a qualitative approach. The study employs interviews with professionals engaged in DV victim support, encompassing both DV and Commissions for the Protection of Children and Youth (CPCJ) professionals. The selection of this methodology is grounded in its congruence with the study’s primary objectives.

### Participants

In Portugal, the RNAVVD plays a vital role in supporting DV victims. This network comprises victim support centers and shelters, overseen by interdisciplinary teams, responsible for conducting comprehensive risk assessments and risk management for victims, addressing individualized needs, devising personalized intervention strategies, and continuously enhancing safety protocols. Additionally, there are CPCJ, responsible for promoting and safeguarding the rights of children and youth. In recent years, complaints of DV have been the primary risk factor reported to the CPCJ. Consequently, all these professionals possess extensive experience in the field of DV, rendering them privileged informants and experts in dealing with DV victims.

The study comprised 24 DV professionals who have been actively involved in providing direct support to DV victims since at least March 2020, coinciding with the outbreak of the COVID-19 pandemic. The sample size was determined based on data richness and depth, and data collection concluded when thematic saturation was achieved, and no new themes emerged. Among the 24 participants, aged between 26 and 60 years, only 2 were male. Their educational backgrounds were diverse, with a significant portion having studied psychology (41.6%). In terms of academic qualifications, 54.2% held a bachelor’s degree. Participants’ experience in working with DV victims varied from 2 to 23 years. Additional sociodemographic characteristics are presented in Table 1.

**Table 1.** Participants’ Sociodemographic Characteristics.

Baseline Characteristic	*N*		%
Gender			
Female	22		91.7
Male	2		8.3
Highest educational level			
Bachelor’s degree	13		54.2
Master’s degree	9		37.5
PhD degree	2		8.3
Training area			
Education	2		8.3
Social/educational service	7		29.2
Nursing	1		4.2
Criminology	1		4.2
Psychology	10		41.6
Sociology	3		12.5
Years worked in domestic violence			
2–5 Years	11		45.83
6–10 Years	6		25
11–15 Years	4		16.67
>16 Years	3		12.5
Age (in years)	Mean = 42.6	*SD* = 7.9	Range: 36–60

*Note.* Participants worked on average 8.7 years in domestic violence (*SD* = 6.1).

### Instruments

This study utilized two data collection instruments: a sociodemographic questionnaire and a semi-structured interview guide. The questionnaire covered participants’ age, sex, educational qualifications, and professional experience, including academic training and years of experience working with DV victims. The semi-structured interview guide underwent careful development and refinement through a preliminary test with a DV professional. After this testing, the interview protocol required no further adjustments. The semi-structured interview consisted of seven open-ended questions organized into four sections. The initial part explored challenges and difficulties encountered during intervention with DV victims. With these goals, participants were queried: “Reflecting on your professional experience with DV victims, what do you consider to be important aspects for working in this field?” and “What are the major challenges in working with DV victims?.”

The second section investigated the impact of exposure to victims’ suffering on professionals’ well-being and their coping mechanisms. Inside this set, the questions were delineated as: “What do you think about the potential impact on your mental health of working with DV victims?” and “What strategies do you employ to cope with the potential impact you described?.”

The third examined the effects of the COVID-19 pandemic and related measures on professionals’ mental health and self-care practices. In this section, the questions were as follows: “How has the COVID-19 pandemic and containment measures affected your life?” and “What strategies did you use in order to foster your well-being during this pandemic?.”

Finally, the fourth section sought to derive valuable guidelines and recommendations for upcoming DV workers, aiming to improve practices and foster emotional and psychological equilibrium. The open question posed was as follows: “Reflecting on the importance of maintaining emotional and psychological balance, what guidance would you offer to a colleague who is embarking on a career in working with domestic violence victims?.”

Analysis primarily focused on data collected from the first and fourth sections due to their extensive content and data richness. Data from the remaining sections will be addressed in distinct publications, considering distinct theoretical considerations.

### Procedures

This research study obtained ethical approval from the University of Maia Ethics and Deontology Council. Access to potential participants was facilitated by contacting the Portuguese RNAVVD and the CPCJ, with formal requests for authorization directed to these institutions. Correspondence with the institutions was conducted via email, providing relevant information about the research project and its objectives. Professionals received an information leaflet outlining the study’s purpose, and those interested in participating were invited to contact the researcher voluntarily. After expressing interest, each professional was contacted via email, and a meeting was scheduled with a project researcher. This meeting could be conducted remotely through a video call platform or in person. During the meeting, all necessary clarifications were provided and informed consent was obtained.

### Data Collection

The data collection phase involved conducting a total of 24 interviews, with 10 conducted via videoconference and 14 in person. The interviews were primarily administered by the first author, an experienced professional with expertise in victim intervention, affiliated with both the RNAVVD and the CPCJ, and holding a Master’s degree in Psychology. Additionally, two well-trained master’s students in Psychology conducted eight of the interviews. The interviews were conducted in Portuguese, between February and October 2022, ranging from approximately 10 to 55 min, with an average of 25 min and a *SD* of 13.4.

### Data Analysis

The research interviews were recorded and transcribed to accurately represent the data. Data analysis and coding were performed collaboratively by the first author and a master’s student with specialized training in the research domain. An inductive, thematic analysis methodology, guided by Braun and Clarke’s (2006) guidelines and supported by the NVivo 10 Qualitative Data Analysis Program, was adopted for the analysis.

To ensure coding process reliability, the two coders independently familiarized themselves with the data by thoroughly reading and re-reading all the interview transcripts. The initial two interviews were jointly coded to establish themes and subthemes. Subsequently, both coders individually coded the remaining interviews, and later, they convened to review and achieve consensus on the final set of themes and subthemes. Additionally, a senior researcher (last author) conducted an audit of the coding process to ensure rigor and validity.

## Results

The study’s findings revealed three main themes describing the experiences and recommendations of DV professionals, with each main theme further divided into subthemes (see Table 2). Throughout the text, direct quotations from the interview transcripts highlighting specific aspects of these themes are included and identified by an interviewer code. Participants’ quotes are presented in English, and a rigorous process of translation was undertaken to ensure the preservation of the original meanings of the participants’ narratives.

**Table 2.** Codification Grid.

Theme	Subtheme	*N*
Challenges in the intervention with DV victims	Complexity of the intervention	22
	System constraints	16
	Victim’s vulnerabilities	23
Difficulties caused by the COVID-19 pandemic	Redefinition of intervention methodologies	17
	Increased stressors	17
Guidelines for future DV workers	Personal skills	21
	Technical skills	24

*Note.* DV = domestic violence.

### Theme 1: Challenges in the Intervention with DV Victims

#### Complexity of the Intervention

Numerous participants (*n* = 20) underscored the complexity inherent in intervening in cases of DV, largely attributed to the myriad of needs presented by victims across diverse domains such as housing, finances, education, employment, and health. They stated that this intricate interplay of needs complicates both the diagnostic process and the subsequent formulation of an individualized intervention plan. Moreover, participants emphasized that these challenges are further exacerbated when professionals lack a comprehensive theoretical understanding of the subject matter and a nuanced awareness of the available resources within the given territory.

Almost one out of three participants (*n* = 7) find that the complexity of DV is apparent in the risk assessment process, which involves a careful analysis of the potential dangers and threats to determine the likelihood of violence escalation. As a result, participants mentioned that formulating a safety plan with personalized and proactive strategies to help the victim protect herself and her children from potential aggressions is difficult.

The unpredictability of the scenario encountered when attending to victims poses another significant challenge for some participants (*n* = 10), as they lack prior knowledge about the specific circumstances each victim will present. Participants believe that this uncertainty imbues the intervention with an unpredictable nature, demanding a constant need for an adaptive and flexible approach to address the unique needs of each victim.

Moreover, a large number (*n* = 17) emphasized the difficulty of listening to the victims’ stories and being directly exposed to their suffering, which resulted in a negative impact on their own emotional and psychological well-being. One participant reported that “I believe that at times, professionals find it challenging to separate what they hear from their personal lives, especially when the narratives they encounter are emotionally heavy and difficult to process” (p. 15).

Additionally, adopting an empathetic and respectful stance toward the victim proved complex for a significant portion (*n* = 16), as they acknowledged the difficulty of fully empathizing with the victim’s circumstances. Some participants also acknowledged struggles in accepting the victim’s narrative and decisions without imposing value judgments, especially when such decisions may involve heightened risk. Notably, certain professionals (*n* = 5) in the child protection system emphasized the heightened challenges in balancing the needs of the victim while safeguarding the best interests of the child. As elucidated by one participant: “There is a need to comprehend the family dynamics and their protective capacity, even within the context of domestic violence. The focus remains on how the family, amidst crisis, manages to organize itself to ensure the well-being of the children” (p. 6).

#### System Constraints

Half of professionals (*n* = 12) highlighted the recurring issue of insufficient support for DV victims. They identify a lack of resources to address various needs, including housing, economic support, employment, and resources for children. The absence of these responses frequently traps victims in abusive relationships, as they struggle to afford basic necessities like housing, food, and childcare. Furthermore, they also highlight the insufficient resources in healthcare, especially regarding mental health, considering the emotional and psychological consequences that victims face due to their history of victimization. Consequently, participants express difficulty in providing tailored solutions to meet the victims’ needs. For them, this obstacle significantly hinders their ability to empower and enable victims to achieve autonomy, further complicating victims’ ability to leave abusive relationships.

Some participants (*n* = 5) expressed concern about the lack of specific responses to address cases of elder abuse. They argued that elderly individuals face unique challenges due to their advanced age, health issues, and often dependence on others, rendering them more susceptible to abuse. Additionally, they emphasized that, in many instances, the perpetrators are the elderly individuals’ own adult children, who also serve as their primary caregivers. This complex dynamic makes it exceedingly difficult for the elderly to report abuse. On one hand, they fear the legal consequences that may befall their adult children, while, on the other hand, they grapple with the stark reality of not having a place to live, coupled with the absence of essential physical and emotional care. They also observed that short-term solutions, like temporary shelters for DV victims, are insufficient in addressing the long-term safety, housing, and emotional support requirements of the elderly. Additionally, they emphasized the problem of limited availability of spaces in nursing homes, leading to prolonged waiting periods for elderly victims seeking a secure and appropriate living environment. One participant stated “When elderly individuals are dependent on the care of others, and the alleged perpetrator is indeed the caregiver, it becomes very complicated to provide adequate responses! Nursing home vacancies are scarce, and therefore, this has been my main challenge in intervening with victims” (p. 16).

A group of participants (*n* = 6) also highlighted the lethargy of the judicial system, noting prolonged legal proceedings and advocating for expedited implementation of measures to protect victims and prosecute aggressors. They also stressed inadequate resources for effectively intervening with aggressors, which they figure as essential for promoting their reeducation and behavioral change, as well as providing therapeutic interventions for underlying issues such as control problems, impulsivity, or substance abuse. Participants further pointed out that the absence of such interventions leads to a recurrence of violent behavior, not only with the same victim but also targeting new victims in subsequent relationships. One participant said “There cannot be a quality intervention in mitigating and slowing down DV without intervening with the aggressor (. . .) It’s a matter of education and involving them in the awareness process . . . because otherwise, that aggressor won’t understand what happened, will always blame the victim, and will continue to be violent even after the relationship ends . . . they will continue in subsequent relationships . . .” (p. 2). Additionally, two participants highlighted that victims frequently seek intervention for the aggressors independently, with the goal of ending the cycle of violence while maintaining their relationships.

One out of three (*n* = 8) emphasized the inadequacy of support available to professionals in the context of DV intervention. They highlighted the lack of opportunities for peer sharing and collaboration, insufficient supervision from higher authorities within the agency, and inadequate provision of psychological support for the professionals themselves. For the participants, these deficiencies in support systems negatively impact their well-being and effectiveness in addressing DV cases. One of the participants expressed, “It’s a pity we don’t have psychologists for us. . . unfortunately, I think that is one of the things that we lack— having support for us because running a support office for victims of crime turns out to be a bit exhausting!” (p. 22). Additionally, two participants believe that there is a lack of protection for them, as they are sometimes confronted by the aggressors, leading to a feeling of insecurity, as mentioned by this participant “I honestly think that the system does not provide much protection for us. It really doesn’t protect us at all . . . there is no protection for the professionals . . . nor for the victims, let alone for the professionals” (p. 13).

Furthermore, seven participants highlighted the challenges that arise when navigating bureaucracies and establishing effective communication with other institutions, emphasizing that other professionals often fail to understand the dynamics associated with DV. Some of the participants also mentioned the legitimization of violence as a difficulty, which contributes to a culture of silence and tolerance, creating additional barriers for victims seeking support and protection. They revealed that there is still a devaluation of violence, not only by the community but also by other professionals and institutions, which negatively affects the appropriate support and intervention with victims.

#### Victim Vulnerabilities

Many professionals (*n* = 19) highlighted challenges centered around the victims, particularly in relation to their vulnerabilities. They noted that when victims seek support, they often exhibit a variety of vulnerabilities, such as a high risk of experiencing further aggression, dependency, emotional and psychological instability, ambivalence toward the aggressor, and a history of tumultuous life experiences. For these participants, these factors collectively contribute to the complexity of the intervention process. One participant mentioned: “Dealing with people’s emotions is even more challenging; they arrive in a very fragile state, not knowing what to do . . . Typically, they come with many years of suffering that few people are aware of, and they are extremely fragile . . .” (p. 8). Another participant highlighted the risk faced by victims, saying that “The prospect of someone being with us while we are aware that their life is in danger is truly frightening” (p. 3).

The majority (*n* = 20) expressed the instability in the victims’ decision-making process and their tendency to excuse or justify abusive behavior. One participant stated, “The main challenge is the victims’ resistance to change, as they often come up with excuses to reconcile” (p. 8).

### Theme 2: Difficulties Caused by the COVID-19 Pandemic

#### Redefinition of Intervention Methodologies

Although not originally included in the interview script, the challenges posed by the COVID-19 pandemic and the imposed restrictions emerged as a prominent theme in the participants’ discourse. Many participants (*n* = 17) noted that the pandemic brought new difficulties to their intervention with victims. Due to the imposed restrictive measures, many participants had to shift to remote work from home, where they did not always have the necessary conditions to provide tailored interventions. Consequently, they noted the necessity of redefining their intervention approach, which in turn introduced further complexities.

The decrease in f2f interactions led to the adoption of remote interventions, including video calls and phone calls. As a result, participants reported facing greater difficulties in establishing a trusting relationship with the victims, as stated by this participant: “The fact that we are not physically present with the victims is a significant barrier because they do not know who they are talking to; they cannot see, and it is a very different experience . . . It becomes a big difficulty for them to trust” (p. 16).

On the other hand, some participants mentioned that not all victims were receptive to this type of remote intervention, as some had limited digital literacy, while others lived in rural areas with internet service issues. One participant stated that “The difficulty I experienced was primarily related to psychological intervention. It was challenging to reach the victims, especially in this area, as it is more rural, and people were not very receptive to contact via WhatsApp or video calls . . .” (p. 21).

Furthermore, nearly half of the participants (*n* = 10) found it challenging to manage contact with victims who were confined at home with their abusers. This circumstance hindered the establishment of communication with the victims and the assessment of their situations. Participants noted that the violence was more hidden, and it was more difficult for the victims to talk about their safety and the abuse suffered. As a result, a few professionals (*n* = 3) mentioned resorting to alternative strategies during their contact with victims, such as pretending to be professionals reaching out to assess economic or health-related situations.

#### Increased Stressors

Several participants (*n* = 17) highlighted the emergence of unforeseen and intensified challenges during the COVID-19 pandemic. Some reported an increase in demands and workload, often accompanied by the addition of new responsibilities, leading to significant physical and psychological exhaustion. One participant elucidated that “Clearly, this has created a higher demand for response and increased distress in the absence of it . . . adding to all of this, there’s been significant strain due to the workload overload during the pandemic” (p. 24). Another mentioned, “As a professional, I must confess it was a bit more complicated because at that time, everyone was working remotely, and I found it stressful because the cases kept coming in, and I couldn’t handle them at the same pace as I’m used to working in person” (p. 8).

Furthermore, due to working remotely, some participants found it challenging to access support and guidance from professional colleagues. They also encountered greater difficulties in accessing resources to meet the needs of the victims, as all institutions were reorganizing in response to pandemic-imposed restrictions. Moreover, when in-person interventions were unavoidable, participants expressed apprehension about the potential for contagion and subsequent transmission of the virus to their families.

For half of the participants (*n* = 12), remote work also resulted in greater difficulty balancing professional and family demands. Some encountered the dual role of fulfilling work obligations while assisting their children with distance learning, while others struggled to maintain a clear boundary between work and personal life and achieve a balance between their professional and personal demands. In this regard, one participant remarked, “I couldn’t distance myself . . . work from home brought us more difficulties . . . we were working, we were taking care of our children, and we thought we had to be working all the time . . .” (p. 18).

Furthermore, numerous participants (*n* = 16) mentioned experiencing a negative impact on their emotional and psychological well-being, reporting symptoms such as anxiety, sadness, and fatigue. The impact of the pandemic on participants’ mental health, as well as the self-care strategies employed during this time, will be subject to analysis in a future publication.

### Theme 3: Guidelines for Future DV Workers

#### Personal Skills

Some participants (*n* = 6) emphasized the significance of self-awareness among professionals, acknowledging its role in identifying areas for personal growth and recognizing potential factors that might adversely affect their intervention methods. One participant specifically highlighted this aspect by stating, “. . . get to know yourself, get to know yourself minimally as a person, get to know yourself as a professional . . . and also realize which are the areas that eventually you don’t control or that you don’t have the necessary knowledge” (p. 6). Other participants reported that the professional’s resilience is considered crucial for effectively managing the demands and occasional frustrations experienced while working with victims.

The significance of self-care was frequently emphasized by nearly half of the participants (*n* = 10) as an essential skill, deemed crucial not only for the well-being of the professional but also for enhancing the quality of intervention with the victim. Among the primary forms of self-care mentioned by participants were maintaining a balance between professional and personal life, nurturing relationships of affective closeness, employing cognitive strategies like abstraction, fostering a positive attitude toward life, engaging in physical exercise, pursuing gratifying activities, seeking social support, and practicing relaxation or mindfulness techniques. Additionally, some participants highlighted the importance of emotional distancing from the victim’s narrative as another aspect of self-care to achieve greater emotional equilibrium.

#### Technical Skills

The development of technical skills emerged prominently in the participants’ speeches, focusing primarily on methodological aspects aimed at enhancing the quality of intervention while concurrently empowering the victims. Consequently, the necessity for specialized knowledge and training in the field of DV was underscored by several participants (*n* = 11), with recognition that it is a continuous learning process, where practical experience holds significance. They emphasized the value of a strong theoretical foundation in victimology and victims’ intervention to remain updated on the intricacies of the intervention process, pertinent legal considerations, and the availability of local social resources. One participant emphasized, “The first piece of advice is a lot of training, a lot of theoretical support with an understanding of the victimization phenomena, an understanding of how victims work” (p. 20).

Several participants (*n* = 18) highlighted the necessity for professionals to possess competence in empowering victims and fostering their safety and autonomy. Additionally, about a dozen (*n* = 12) emphasized the importance of professionals being capable of managing expectations and recognizing the boundaries of their intervention. As one participant stated, “I advise you to reduce expectations . . . understand what you can really do, what it’s real, what it’s within reach . . .” (p. 20).

Empathetic, non-judgmental, and respectful attitudes were emphasized as crucial guiding principles in the intervention with victims by a significant number of participants (*n* = 15). These professionals reported encountering situations where the decisions made by victims might not align with their own recommendations, leading to feelings of frustration and dissatisfaction. Nonetheless, it was strongly emphasized that the autonomy to make decisions should always lie with the victim, and professionals must consistently uphold and respect these choices, irrespective of any emotional impact it may elicit. As expressed by one participant, “the decision always rests with the victim, and the future professional has to know how to respect her decision despite the frustration it may cause him” (p. 17).

Lastly, some participants (*n* = 9) underscored the value of seeking supervision from experienced professionals or supervisor and receiving support from colleagues. This entails sharing experiences, expressing emotions, analyzing intervention strategies, and seeking assistance in decision-making processes, as exemplified in the following statement: “. . . making decisions isn’t easy, and venting helps because we’re human too. We acknowledge the possibility of making mistakes, as nobody is perfect. If we can reduce these chances, it is even better!” (p. 21).

## Discussion

The present study focused on investigating the perspectives and experiences of professionals engaged in DV intervention. The research aimed to address the existing gaps in the field, examining both their routine practices and the additional challenges encountered during the pandemic, in the Portuguese context. The qualitative nature of the present study allowed to uncover professionals’ narratives and voices, when dealing with the complexities of intervening with DV victims, especially in the context of COVID-19 pandemic. As difficulties brought by the pandemic crisis are mentioned in previous literature, no study, to our knowledge, also discusses the narratives of the complexity of intervening with DV victims and also some guidelines on how to do it healthily, from the lens of the professionals.

Taking a closer look at the challenges of DV intervention, participants in this study acknowledged risk assessment and safety planning to be particularly complex. The study highlights the inherent difficulty of evaluating the numerous factors that impact the safety of victims, even for experienced professionals. Aligned with prior research (Burnett et al., 2016; Frieze et al., 2020; Fusco, 2013; Merchant & Whiting, 2015), this study underscored challenges tied to maintaining empathy, addressing victims’ vulnerabilities, navigating the instability of the change process, the ambivalence toward the aggressor and managing the frustration stemming from victims’ decisions to remain in or return to abusive relationships.

The study revealed a clear emotional impact on professionals, arising from their engagement with victims’ accounts and significantly affecting their well-being. These findings are consistent with other studies indicating that DV professionals experience elevated stress levels resulting from both direct and indirect exposure to victims’ narratives and suffering (Kulkarni et al., 2013), as well as other negative psychological outcomes (Ellis & Knight, 2021).

Although prior research has addressed similar challenges, this study unveils novel aspects that warrant further exploration. The results revealed that participants acknowledged the intricate nature of intervening in DV cases, recognizing the diverse needs of victims across various domains. While previous authors have discussed the importance of evaluating and responding to victims’ diverse needs to empower them (Sullivan & Goodman, 2019), our study uncovers additional challenges beyond the necessity for professionals to possess knowledge, training, and resource mobilization skills. This research underscores that the challenge of meeting victims’ needs can be further compounded by insufficient institutional solutions for DV victims. This finding warrants careful consideration, as inadequate resources may compromise intervention effectiveness, preventing professionals from providing necessary support for victims to break free from the cycle of violence. Moreover, insufficient guidance or unrealistic expectations about available resources may create a false sense of security in victims, leading to hasty decisions that may expose them to greater social vulnerability.

The challenge of meeting victims’ needs becomes even more pronounced when considering elderly victims. The study highlighted their unique vulnerabilities, including advanced age, health issues, and dependency, which increase their susceptibility to abuse. Professionals also observed that perpetrators often include the victims’ own adult children, hindering the reporting process. Additionally, the study identified significant gaps in existing interventions, particularly in providing solutions that ensure housing and care for elderly DV victims. Temporary shelters fail to adequately address the long-term safety, housing, and emotional support needs of the elderly, while insufficient space in nursing homes results in prolonged waiting periods for suitable accommodation.

A closer examination of the study’s results also reveals that intervention challenges extend beyond direct assistance to victims. The findings emphasize the perceived need, not only among professionals but also among victims themselves, for targeted interventions specifically aimed at aggressors. These interventions aim to prevent violence recurrence through reeducation and behavior modification. While professionals can empower and support victims, the absence of interventions designed to work with and rehabilitate aggressors may perpetuate the cycle of violence, impacting not only current victims but also potential future ones. Additionally, professionals acknowledge that sometimes victims themselves seek help from the aggressor, despite a history of abuse, in hopes of maintaining the relationship provided the violence ceases.

The previously discussed challenges, compounded by a perceived lethargic judicial system in deciding DV cases, are further exacerbated by the difficulties professionals encounter in accessing support, supervision, and psychological assistance. This situation leads to feelings of insecurity and frustration. Similar to the present study, the literature has pointed out that alongside the lack of supervision and peer sharing, bureaucracy and the cultural legitimization of violence are factors that create obstacles to intervention with victims (Frieze et al., 2020; Kulkarni et al., 2013; Lamothe et al, 2018; Radey & Stanley, 2018).

The COVID-19 pandemic brought forth significant and cumulative challenges for professionals working with DV victims. The shift to remote work required a rethinking of intervention methods, mostly conducted at a distance, leading to challenges in building trust and communicating with victims at home with their abusers, worsened by issues like limited digital skills or internet access. These challenges resonate with prior research (Caridade et al., 2021; Garcia et al., 2022; van Gelder et al., 2021; Voth Schrag et al., 2023), indicating a broader trend in the field.

Additionally, our findings highlighted several challenges like increased burden and stress due to overlapping family and professional responsibilities (Self-Brown et al., 2022), difficulties in maintaining work-home balance and boundaries (Voth Schrag et al., 2023; Wood et al., 2022), social isolation from significant others (van Gelder et al., 2021), and fear of contamination during in-person interventions (Self-Brown et al., 2022). Overall, professionals reported a deterioration of their mental health during the pandemic, encompassing feelings of frustration, insecurity, and loneliness, as well as anxiety, stress, depression, and worry. These findings emphasize the substantial psychological strain endured by professionals during the pandemic, a trend consistent with observations from other studies (Self-Brown et al., 2022; van Gelder et al., 2021).

It seems undeniable that intervening with DV victims in challenging contexts, particularly those imposed by the COVID-19 pandemic demands a reevaluation of professionals’ skills and working conditions to ensure effective support. The guidelines derived from the participants’ discourses align with existing literature, even prior to the pandemic, emphasizing the importance of personal and technical skills for DV professionals (Caridade & Sani, 2018; Illescas, 2016). Notably, self-awareness is considered a fundamental personal skill, enabling them to assess their thoughts, feelings, and behaviors, while also recognizing biases and cultural influences (Sullivan & Goodman, 2019; Viskovich & De George-Walker, 2019). Another significant guideline is the practice of self-care, ensuring their well-being and effective support for victims (Viskovich & De George-Walker, 2019). Other studies emphasize its role in reducing emotional distress and preventing burnout (Salloum et al., 2015).

Technical skills are crucial for DV professionals, as Fusco’s (2013) findings on the importance of education and training in DV issues illustrated. Adequate training enhances professionals’ abilities to respond to victims’ needs, validate disclosures, and avoid biased attitudes (Langenderfer-Magruder et al., 2019). It is imperative to sustain empathy, non-judgment, and respect for victims’ decisions. Continuous education empowers professionals to effectively assist victims, improve risk assessment, and acquire new knowledge (Frieze et al., 2020). Professionals engaged in collaboration with peers require skills in negotiation, conflict resolution, assertiveness, and effective communication (Sullivan & Goodman, 2019). Lastly, supervision and peer support are fundamental aspects. Sharing experiences and strategies with colleagues not only enriches interventions but also serves as a coping and self-care mechanism for professionals (Lamothe et al., 2018).

Especially during crisis scenarios like the COVID-19 pandemic, additional guidelines take on significant importance, as professionals pointed out. It is crucial to address digital literacy gaps among victims and provide training in online tools and virtual support for professionals (Caridade et al., 2021; van Gelder et al., 2021). Professionals need the ability to assess risk remotely, especially when victims are confined with the perpetrator, and to implement measures to improve victims’ safety (Self-Brown et al., 2022). They should also be ready for crisis situations and integrate self-care strategies into their routines to manage stress and maintain a healthy work-life balance (Macy, 2022; van Gelder et al., 2021; Voth Schrag et al., 2023). As noted by Self-Brown et al. (2022), is crucial to implement mechanisms to facilitate better organization for professionals and their families during potential crisis situations. While it is important to implement social policies to mitigate the disparities victims face in remote interventions (Ghidei et al., 2022), it is equally crucial to direct specific measures toward professionals that can effectively alleviate fatigue and burnout, such as providing psychological support, online coaching, and supervision (Ghidei et al., 2022; van Gelder et al., 2021).

## Limitations and Conclusions

This study uncovers the significant challenges encountered by professionals supporting DV victims, especially during crises like the COVID-19 pandemic. By delving into these complexities, the research offers valuable contributions to improving DV intervention strategies, especially in crisis scenarios. While focusing on the Portuguese context, the findings have global relevance, underscoring the demanding nature of assisting DV victims and the need for organizations and professionals to address the diverse obstacles.

One limitation regards demographic representation, with only two male participants. Although there is no trustworthy information about it, it is our belief that most of the professionals who work with DV in Portugal are probably women. While the sample includes DV professionals from various agencies in Portugal, a broader recruitment strategy could have ensured a more diverse geographical representation.

The results highlight the urgency for increased investment in public policies to support DV victims, addressing not only housing and economic issues but also their physical and mental health needs. Special attention to elderly victims is crucial, given the inadequacy of traditional shelters and limited vacancies in nursing homes. Moreover, the study underscores the significance of intervening with aggressors alongside providing support to victims. Without adequate intervention to address behavior change, substance abuse, and impulsivity, as well as to prevent recidivism, DV intervention measures may fall short.

The complexity of the challenges in intervening with DV victims, especially in crisis scenarios, highlights the psychological vulnerability of the professionals involved. Therefore, an important line of investigation may involve evaluating the impact that intervening with victims has on the mental health of professionals, further exploring the role of coping and self-care strategies.

This study has also contributed to the definition of guidelines for DV professionals, highlighting the importance of technical skills, self-awareness, and practicing regular self-care. Organizations should review intervention policies and working conditions, promoting continuous training, supervision, and collaboration among peers. Training should include adapting to crisis contexts, such as establishing remote relationships with victims and conducting risk assessments from a distance. Organizations should also create a mental health culture that supports professionals and mitigates burnout, providing psychological support and implementing policies that encourage self-care, such as flexible hours, training on self-care, and stress management. In crisis contexts, supervision and peer support should be provided remotely, along with psychological support resources.

In conclusion, this study emphasizes the pressing need for comprehensive strategies to address the multifaceted challenges faced by professionals supporting DV victims. Recognizing the global significance of these findings and advocating for enhanced support systems can help strive for a more effective response to DV, safeguarding the well-being of both victims and dedicated professionals.
